# Mixed radiation with different doses induces CCL17 to recruit CD8^+^T cell to exert anti-tumor effects in non-small cell lung cancer

**DOI:** 10.3389/fimmu.2024.1508007

**Published:** 2025-01-14

**Authors:** Liuying Huang, Duo Wang, Muchen Xu, Danqi Qian, Yulin Cao, Xiaohan Wu, Liang Ming, Junhui Tang, Zhaohui Huang, Yuan Yin, Leyuan Zhou

**Affiliations:** ^1^ Department of Radiation Oncology, Affiliated Hospital of Jiangnan University, Wuxi, China; ^2^ Department of Radiation Oncology, The Fourth Affiliated Hospital of Soochow University, Suzhou, China; ^3^ Wuxi Cancer Institute, Affiliated Hospital of Jiangnan University, Wuxi, China; ^4^ State Key Laboratory of Radiation Medicine and Protection, Soochow University, Suzhou, China

**Keywords:** lung cancer, radiotherapy, animal research, immunotherapy, combination therapy

## Abstract

**Background:**

Different doses of radiotherapy (RT) exert diverse effects on tumor immunity, although the precise irradiation method remains unknown. This study sought to elucidate the influence of combining different doses of RT with immune checkpoint inhibitors (ICIs) on the infiltration of CD8^+^T cells within tumors, thereby augmenting the anti-tumor response.

**Methods:**

Constructing a mouse model featuring bilateral lung cancer tumors subjected to high and low dose irradiation, the analysis of RNA transcriptome sequencing data and immunohistochemical validation for tumors exposed to various dosages guided the selection of the optimal low-dose irradiation scheme. Subsequently, upon the integration of immune checkpoint inhibitors (ICIs) therapy, the infiltration of immune cells within the tumor was ascertained via immunohistochemistry (IHC) and flow cytometry (FCM). Finally, through bioinformatics analysis and experimental verification, potential strategies to bolster the anti-tumor immune response were investigated.

**Results:**

In comparison to the administration of 20Gy alone to the primary tumor, supplementing with 6Gy directed at the abscopal tumor produces a more pronounced abscopal response. The synergy of 20Gy, 6Gy, and ICIs markedly boosts the efficiency of ICIs. According to the findings from IHC and FCM studies, the triple therapy group exhibits a heightened infiltration of immune cells into the tumor, largely attributable to the augmented expression of CCL17 within the tumor under these irradiation regimens, which subsequently draws CD8+ T cells to infiltrate the tumor site, exerting cytotoxic effects.

**Conclusion:**

Our study shows that the combined application of 20Gy and 6Gy can enhance the infiltration of tumor CD8^+^T cells in mice and improve the effectiveness of immunotherapy.

## Introduction

Approximately 70% of cancer patients receive radiation therapy, which is regarded as one of the primary treatment modalities for cancer (1). Radiotherapy (RT) primarily exerts its therapeutic effect by inducing acute DNA damage, which triggers apoptosis or other forms of cell death in cancer cells (2). Technological advancements in RT, including four-dimensional computed tomography (4DCT), intensity-modulated RT (IMRT), respiratory gating techniques, and stereotactic ablative body RT (SABR), have facilitated higher tumor doses while reducing exposure to surrounding tissues (3). Consequently, an increasing number of patients are benefiting from these improvements (4). However, the efficacy of RT as a localized treatment remains limited, thereby constraining its application in the management of metastatic illness. In 1953, Mole and his colleagues were the first to define radiation-induced systemic antitumor effects that could shrink tumor lesions in irradiated areas and unirradiated regions (5). This phenomenon, known as the abscopal effect, offers a novel therapeutic approach for managing metastatic diseases, such as lung cancer that has spread to the brain, bone, adrenal glands, and face, as well as areas unsuitable for high doses of radiation therapy. Nevertheless, reports of distant effects caused by radiation therapy alone remain rare (6).

These observed medical phenomena prompted a search for methods to enhance the far-reaching effects of radiation therapy. In recent years, there has been increasing emphasis on the integration of immunotherapy with RT (7). A growing body of clinical trial- and pre-clinical data has demonstrated that immunotherapies significantly improve the radio-induced distancing effect (8–10). Research into the combined use of RT with anti-CTLA-4 and anti-PD-L1 antibodies in melanoma revealed that 26% of cases exhibited this distancing effect (11). While these findings suggest an enhancement of the distancing effect, the benefits appear to be limited to a minority of patients, with the majority still demonstrating poor therapeutic responses. The manifestation of distant effects can be influenced by various factors, including RT dosage and segmentation mode, immunotherapy modalities, the timing of interventions, and tumor type (12, 13). Consequently, it is imperative to explore novel strategies to optimize these combinatorial approaches. Further investigation is warranted to elucidate the mechanisms underlying the variability in treatment responses and to develop more effective therapeutic protocols that can benefit a broader patient population.

High-dose RT (HDRT) has been shown to elicit a vaccination-like response, to alter cancer cell characteristics to enhance their susceptibility to T cell-mediated attack, and to modify the tumor microenvironment to facilitate immune cell infiltration (14). However, for patients with recurrent and metastatic disease, the application of large-field, high-dose irradiation is often impractical. In contrast, low-dose RT (LDRT) plays a crucial role in modulating the tumor-immune microenvironment by promoting immune effector cell infiltration and mitigating the immunosuppressive effects of RT (15). Thus, utilizing a combination of RT regimens may be the optimal approach to induce antitumor immune response.

Lung cancer remains the leading cause of cancer-related mortality worldwide and ranks as the second most prevalent malignancy, with an estimated 2.2 million new cases and 1.8 million fatalities annually (16). Non-small cell lung cancer (NSCLC) accounts for 85% of lung cancer cases, representing the predominant pathological lung cancer subtype (17). Despite advances in treatment, NSCLC-related mortality continues to rise. A significant clinical challenge is that upon initial diagnosis, 75% of NSCLC patients present with metastatic disease, and the five-year survival rate for these patients ranges between 2.8% to 14.6% (18, 19). For patients with advanced stage III or IV lung cancer patients who are ineligible for surgical intervention, the standard treatment regimen comprises combined chemotherapy and RT (20). Over the past decade, the advent of immunotherapy has significantly improved outcomes for locally advanced NSCLC (through consolidation durvalumab following deterministic chemoradiotherapy) and advanced NSCLC (via immune checkpoint inhibitor monotherapy or in combination with chemotherapy) (21, 22). Nonetheless, these approaches have not yielded favorable results in patients with multiple metastases (23). Currently, combination RT and immunotherapy is primarily used in advanced non-metastatic clinical cases, with emerging evidence supporting its efficacy (24, 25). However, the response rate remains limited to 41.7% (26), underscoring the need for further optimization. Consequently, there is an urgent need to explore novel therapeutic strategies that can efficiently integrate RT and immunotherapy, maximizing their anticancer properties.

In this work, we observed several interesting phenomena. When administering a lethal radiation dose to primary metastases in NSCLC patients, we noted that other metastases along the treatment pathway received a lower doses. Subsequently, these patients received immunotherapy. Surprisingly, the metastases that received low-dose radiation showed significant regression following immunotherapy, while other sites remained unchanged. Based on these observations, we established a mixed irradiation model, applying a dose of 20 Gy to one tumor site and a dose less than 20 Gy to another site. Our findings revealed that the combination of 20 Gy and 6 Gy irradiation was optimal, enhancing the efficacy of immune checkpoint inhibitors (ICI) treatment. Specifically, 6 Gy treatment resulted in increased CD8^+^ T-cell infiltration at the tumor site, which correlated with elevated expression of the chemokine, CCL17. Our research demonstrates that the application of this mixed irradiation model significantly enhances the CD8^+^ T-cell infiltration in murine tumors and boost the efficacy of immunotherapy. These findings provide valuable insights into potential strategies for improving the outcomes of combined radiotherapy and immunotherapy in advanced NSCLC patients.

## Materials and methods

### Clinical data

Case 1: A 61-year-old male patient was diagnosed with upper left lung cancer in December 2020. In August 2021, he developed metastases in the nasal region, left face, and right upper limb. Subsequently, in December 2021 the patient started treatment at the Fourth Affiliated Hospital of Soochow University, where he received nasal RT in combination with natalizumab.

Case 2: A 71-year-old male patient presented with a diagnosis of squamous cell carcinoma of the upper lobe of the left lung in May 2024. Positron Emission Tomography-Computed Tomography (PET-CT) imaging revealed increased Fluorodeoxyglucose (FDG) uptake in the left hilum lymph node (station 10), indicating a potential for metastatic involvement. Following the diagnostic workup, the patient underwent Stereotactic Body Radiation Therapy (SBRT) targeting the lesion in the left upper lobe. This was subsequently followed by three cycles of combination therapy, encompassing both chemotherapy and immunotherapy.

Case 3: A 59-year-old male patient was diagnosed with squamous cell carcinoma of the upper lobe of the right lung in July 2024. PET-CT imaging demonstrated increased FDG metabolism in the right upper tracheal lymph node (station 2R), hinting at metastatic involvement. Subsequently, the patient underwent SBRT to address the lesion in the upper lobe of the right lung. This therapeutic intervention was followed by three cycles of chemotherapy combined with immunotherapy.

### Cell lines

The human T lymphocyte Jurkat cell line and the human H1299 NSCLC cell line were purchased from Shanghai Mingjin Biolo Gy Co., Ltd. (Shanghai, China), while the human A549 NSCLC cell line was purchased from the American Type Culture Collection (ATCC). The lines and mouse lung cancer cells (LLC) mouse lung cancer cell line was provided by Nanjing University. LLC and A549 cells were cultured in Dulbecco’s Modified Eagle Medium (DMEM; eallbio, China) containing 10% fetal bovine serum (FBS) and 1% penicillin−streptomycin. Jurkat and H1299 cells were cultured in RPMI 1640 medium (eallbio, China) containing 10% FBS and 1% penicillin−streptomycin. All cells were maintained at 37°C in a humidified atmosphere containing 5% CO_2_.

### Radiation parameters

LLC, H1299, and A549 cells were seeded into T25 culture flasks (Corning, USA). When cell density reached 75%–85%, the cells were exposed to X-ray irradiation doses of 3, 6, 9, and 12 Gy using a linear accelerator (Elekta Limited, Sweden) at a dose rate of 3 Gy per minute. During irradiation, a 1.5-cm-thick bolus was used to adjust the radiation distribution, with the T25 flask positioned on the treatment couch. The radiation properties were as follows: 6-MV photon beam energy, 100 cm source-surface distance, 10×10 cm^2^ radiation field size, and 180° gantry angle. Dosimetry was performed using a cylindrical ionization chamber prior to irradiation.

### Tumor models

Male C57BL/6 mice, aged six weeks and weighing 18 ± 2 g, were obtained from Shanghai SLAC Laboratory Animal Co., Ltd. (Shanghai, China) and maintained in a pathogen-free environment. To establish bilateral tumors, 4×10^6^ LLC cells were injected subcutaneously into the left hind limb to create the primary tumor, while an additional 4×10^6^ LLC tumor cells were implanted in the right limb to form the secondary tumor. When the secondary tumor reached approximately 500 mm^3^ in size, mice bearing bilateral tumors were randomly assigned to various treatment groups.

### Tumor therapy

Tumor progression was monitored and quantified using previously described methods. Prior to radiation therapy, each mouse was shielded with a lead plate to ensure that only the targeted tumor was exposed to radiation. An Elekta Synergy instrument (Elekta limited, Sweden) was used to administer the radiation treatment. An anti-PD-L1 antibody (Bioxcell, USA) was administered via intraperitoneal injection, with an initial dose of 200 µg followed by subsequent doses of 100 µg every three days for a total of five injections. An anti-CTLA4 (Bioxcell, USA) was also administered intraperitoneally at a dose of 100 µg per mouse every three days for a total of five injections. In accordance with ethical standards, mice were euthanized when the implanted tumors reached a volume of 2000 mm^3^.

### CD8A, PD-L1, and CTLA4 mRNA expression in different types of NSCLC in TIMER

The Tumor Immune Estimation Resource (TIMER) database contains 10,897 samples from 32 cancer types sourced from the Cancer Genome Atlas (TCGA). The differential expression of CD8A, PD-L1 (CD274), and CTLA4 between tumors and normal tissues was examined using the TCGA database, and the significance of the difference was determined using the Wilcoxon test. A p-value of less than 0.05 was considered significant.

### Survival analysis of CCL17 in the Kaplan-Mayer plotter database

In this study, we utilized the Kaplan-Meier plotter database to investigate the relationship between the presence of CCL17 expression and survival duration in individuals with lung cancer. The database enables analysis of the impact of 54,000 genes (including mRNA, miRNA, and protein) on survival rates across 21 cancer types. Data for our analysis were obtained from the Gene Expression Omnibus (GEO), European Genome-phenome Archive (EGA), and TCGA databases, which provided comprehensive information. To thoroughly examine the association between CCL17 expression in lung cancer and overall survival (OS), we calculated hazard ratios (HRs) with corresponding 95% confidence intervals (CI) and log-rank P-values (27). This approach allowed for a robust statistical assessment of the prognostic significance of CCL17 in lung cancer.

### CCL17 expression and immune cell correlation in the TISIDB

The Tumor and Immune System Interaction Database (TISIDB) is a comprehensive resource designed to analyze relationships between tumors and various immune components, including tumor-infiltrating lymphocytes (TILs), immunomodulators, and major histocompatibility complexes (MHCs) (28). The TISIDB enables researchers to explore gene functions and their impact on tumor-immune interactions. We employed the Spearman correlation test to evaluate associations between prognostic genes and TILs. All hypothesis tests were conducted using two-sided considerations, with *P* < 0.05 considered statistically significant.

### Flow cytometry

Tumor samples were excised, diced, and then suspended in phosphate buffered saline (PBS) to create a cell suspension. The tumor fragments were then digested with a combination of collagenase, deoxyribonuclease I, and hyaluronidase (Sigma, USA) for 50 minutes at 37°C in a temperature-controlled shaker. The resulting supernatant was filtered through a 70 µm mesh (BD Falcon, USA). Immune cells were isolated using a mouse tumor-infiltrating mononuclear cell isolation kit (Solar Bio, China) and resuspended in PBS at a concentration of 1 × 10^7^/mL. Subsequently, 100 µL of the cell suspension was stained with the fluorescently labeled anti-mouse antibodies: CD3-FITC, CD8-APC (all from Biolegend, USA) for 30 minutes on ice. After two PBS washes, the cells were resuspended for analysis using flow cytometry. These data were analyzed using NovoExpress software (Agilent, USA).

### RNA sequencing analysis

Mice were euthanized by cervical dislocation, and tumors were excised, rinsed twice with DEPC-treated water, minced into small fragments, and transferred to cryostorage tubes. These samples were then stored in liquid nitrogen and sent to Shanghai OE Biotech Co., Ltd. (Shanghai, China) for sequencing. Libraries were sequenced on an Illumina NovaSeq 6000 platform, generating 150 bp paired-end reads. Approximately 50 million raw reads were obtained per sample. Raw reads in FASTQ format were processed using fastp (29) to remove low-quality reads, resulting in approximately 45 million clean reads per sample for subsequent analyses. Clean reads were mapped to the reference genome using HISAT2 (30). FPKM values for each gene were calculated, and read counts were obtained using HTSeq-count (31). Principal Component Analysis (PCA) was performed using R (v3.2.0) to evaluate biological replication of samples. Differential expression analysis was conducted using DESeq2 (32), with a threshold of q-value < 0.05 and fold change > 1 or < 0.5 for significantly differentially expressed genes (DEGs). Hierarchical cluster analysis of DEGs was performed using R (v3.2.0) to illustrate gene expression patterns across different groups and samples. A radar map of the top 30 genes was generated using the R package ggradar to visualize up- and downregulated DEGs. Gene Ontology (GO), KEGG pathway, Reactome, and WikiPathways enrichment analyses of DEGs were performed using R (v3.2.0) based on the hypergeometric distribution to identify significantly enriched terms. Column diagrams, chord diagrams, and bubble plots were generated to visualize significant enrichment terms. Gene Set Enrichment Analysis (GSEA) was conducted using GSEA software (33). This analysis employed predefined gene sets, with genes ranked according to their differential expression between two sample types. The analysis then tested whether the predefined gene sets were enriched at the top or bottom of the ranking list.

### Immunostaining

Tumor samples were fixed in 10% neutral buffered formalin, embedded in paraffin wax, and subsequently stained with hematoxylin and eosin (H&E) or subjected to immunohistochemistry. For immunostaining, tumor sections were deparaffinized, rehydrated, and subjected to antigen retrieval. Sections were then incubated overnight at 4°C with primary antibodies: rabbit anti-CD8 (1:100, Bioss, China), mouse anti-PD-L1 (1:200, Protein, China), mouse anti-CTLA-4 (1:50, Santa Cruz, China), and rabbit anti-CCL17 (1:200, Abcam, UK). Following primary antibody incubation, sections were treated with corresponding secondary antibodies (Genentech, USA). Immunoreactivity was then visualized using diaminobenzidine (DAB) as the chromogen. For quantification, multiple high-power fields were randomly selected from each section, and positively stained cells were counted per unit area.

### ELISA

A549 cells, irradiated and unirradiated at various doses, were resuspended and plated in 24-well plates with 300 µL of complete medium. After 3 days, the supernatant was collected and CCL17 expression levels were evaluated using ELISA kits (Mlbio, China) according to the manufacturer’s instructions.

### qRT−PCR

Total RNA was extracted using TRIzol reagent (TaKaRa, Japan). Complementary DNA (cDNA) was synthesized using the Prime-Script RT reagent kit (CWBIO, China). Radiation-induced mRNA expression was detected using UltraSYBR mixture (CWBIO, China). Actin levels were used as a reference for normalization. The experiment was performed in triplicate, with RT-PCR reactions repeated independently three times. Primer sequences are provided in the **Supplementary Table**.

### Migration assays

Jurkat cells (2 × 10^5^ cells/mL) were plated in 200 μL of serum-free medium (HyClone, USA) in the upper chamber of a 5 µm Transwell insert (Corning, USA). The lower chamber contained 500 μL of medium supplemented with 10% FBS (Albino, China) and either CCL17 chemokines (5 µg/mL) (Sangon, China) or the cell supernatant from irradiated (9 Gy) A549 and H1299 cells, with or without anti-CCL17 mAb (R&D Systems, USA). After a 3-hour incubation, migrated cells were fixed with 4% paraformaldehyde, stained with 0.1% crystal violet for 10 minutes, and washed thrice with PBS. Quantification was performed by analyzing five randomly selected fields.

### Statistical analysis

Data were analyzed using SPSS 22.0 and GraphPad Prism 9.4. Results are expressed as mean ± standard deviation (SD). Two-tailed Student’s t-test was used for comparisons between two groups, while one-way analysis of variance (ANOVA) was employed for comparisons among multiple groups. Results with P < 0.05 were considered statistically significant.

## Results

### Combined RT and immunotherapy induces tumor shrinkage

**Figure 1:**
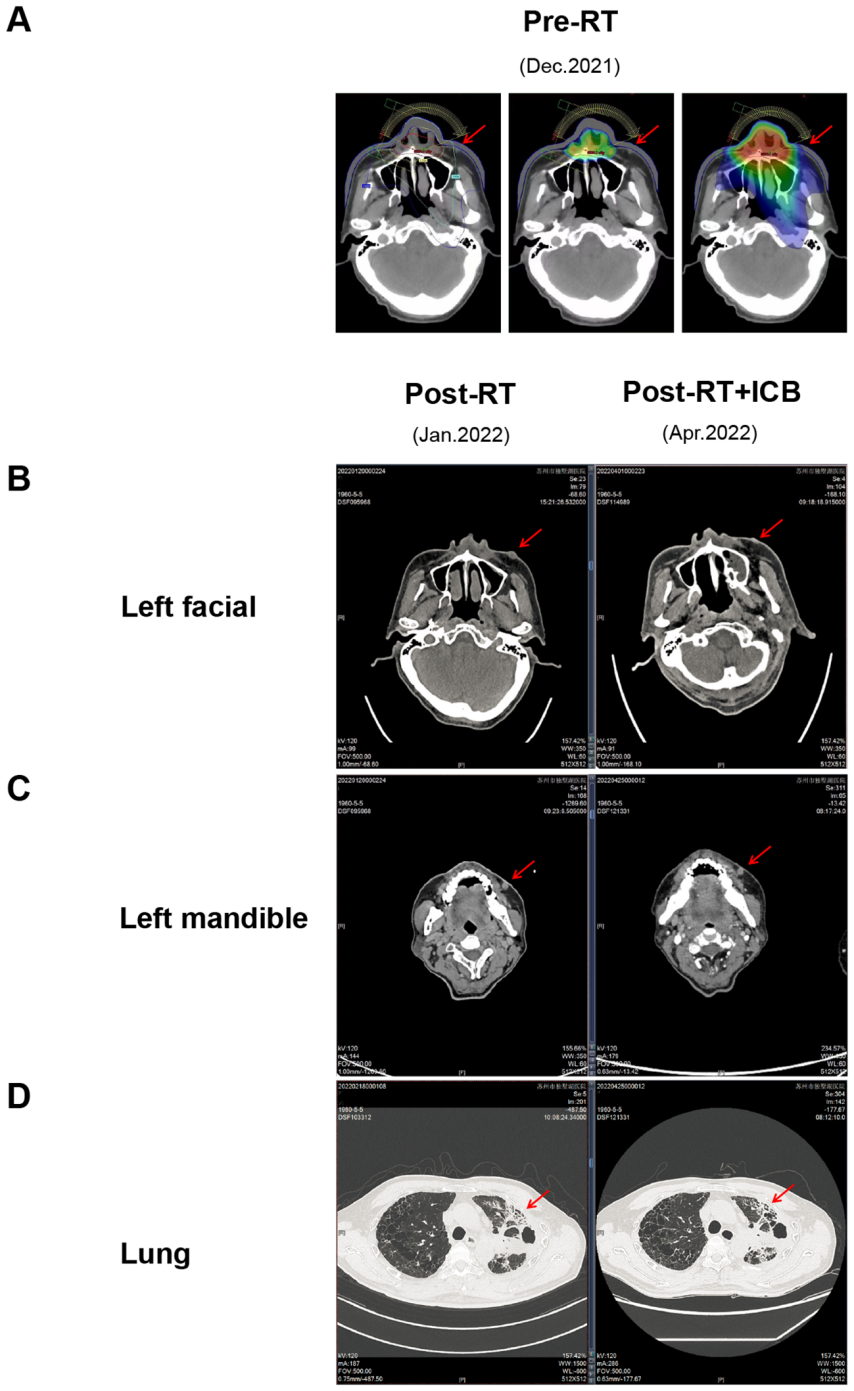
Radiotherapy combined with immunotherapy shrinks the tumor, within the irradiation path. **(A)** Schematic diagram of the irradiation target area,in figure A1, the red dot represents the point with the highest radiation dose at the level of 6324.1cGy, and the yellow, green, and blue lines represent the isodose lines of 5900, 2300, and 1500 cGy, respectively. The radiation dose at each part of the dose line is the value on the dose line, and the radiation dose at the inner part of the dose line is higher than that of the dose line. The radiation dose received outside the dose line was lower than the dose line value. A2 and A3 are dose cloud charts, which intuitively indicate the dose level by color. The middle red part was the highest dose, and the yellow, green and blue irradiation dose decreases layer by layer. The tissues without dose cloud cover indicate that they are not irradiated by radiation.; **(B)** CT images of the left facial mass receiving low-dose irradiation; **(C)** CT images of the left facial mass that did not receive low-dose irradiation; **(D)** CT images of lung lesions. *The red arrow points to tumor lesions.

Case 1 included a 61-year-old man with squamous cell carcinoma of the lung with nasal metastasis. The patient presented to the hospital with primary lung cancer of the left upper lung (December 2020). Later, the patient developed nasal metastasis (August 2021). Nasal radiotherapy (60 Gy/30 f) (December 2021) was performed on the patient. During radiotherapy, the patient’s left face and left jaw developed new tumors from the previous ones (**Figure 1A**). Due to the large irradiation range of the patient’s face, radiotherapy was not performed for the new tumors considering the patient’s facial injuries and other problems. Low-dose radiotherapy (≈20 Gy/30 f) was administered to the left facial tumor in the irradiation path of the nasal tumor, and the nasal tumor significantly shrank after the end of radiotherapy, but the left facial tumor did not shrink. Two cycles of anti-PD-L1 (Etrilizumab) therapy (January 2022) were subsequently performed, and the new tumors on the left face significantly subsided (April 2022) (**Figure 1B**). However, no significant shrinkage was observed in the lung and left mandibular areas that were not irradiated (**Figures 1C, D**).

Case 2 involved a 71-year-old male who was diagnosed with squamous cell carcinoma of the upper left lung lobe in May 2024. PET-CT imaging revealed increased FDG uptake in the left hilar lymph node (station 10), suggesting tumor metastasis to this location (**Supplementary Figure S1A**). Subsequently, in June 2024, the patient underwent SBRT with the upper left lung lobe tumor receiving a dose of 24 Gy/3f, while the adjacent 10L lymph node received a low dose of 5 Gy (**Supplementary Figure S1B**). After completing three cycles of chemotherapy combined with immunotherapy, a CT scan showed a reduction in the size of the upper left lung tumor and alleviation of atelectasis (**Supplementary Figure S2**). Following this, the patient underwent radical surgery, and postoperative pathology confirmed a complete pathological response in the hilar lymph nodes at station 10.

In Case 3, the patient is a 59-year-old male who was diagnosed with squamous cell carcinoma of the upper right lung lobe in July 2024. PET-CT imaging revealed increased FDG metabolism in the right upper paratracheal lymph node (station 2R), suggesting a possibility of metastasis (**Supplementary Figure S3A**). Subsequently, starting from August 2024, the lesion in the upper right lung lobe underwent SBRT with a prescribed dose of 24 Gy delivered in 3 fractions. The adjacent 2R lymph node received a low dose of 5 Gy for the majority of its volume, while the more distant cephalic portion of the lesion received only a dose of 3 Gy (**Supplementary Figure S3B**). Following three cycles of chemotherapy combined with immunotherapy, a CT scan demonstrated a partial response of the tumor lesion (**Supplementary Figure S4**). Later, in October 2024, a lymph node biopsy was performed, and postoperative pathology indicated a complete pathological response in the lymph nodes of station 2R.

These cases suggest that the combination therapy of HDRT + LDRT + ICI may further activate the immune response and enhance anti-tumor efficacy. Therefore, we intend to explore whether this combination therapy mode can be used in some special cases of patients with multiple tumors or multiple metastases that are not suitable for large-area irradiation.

### Mixed-dose radiation therapy inhibits tumor growth

**Figure 2:**
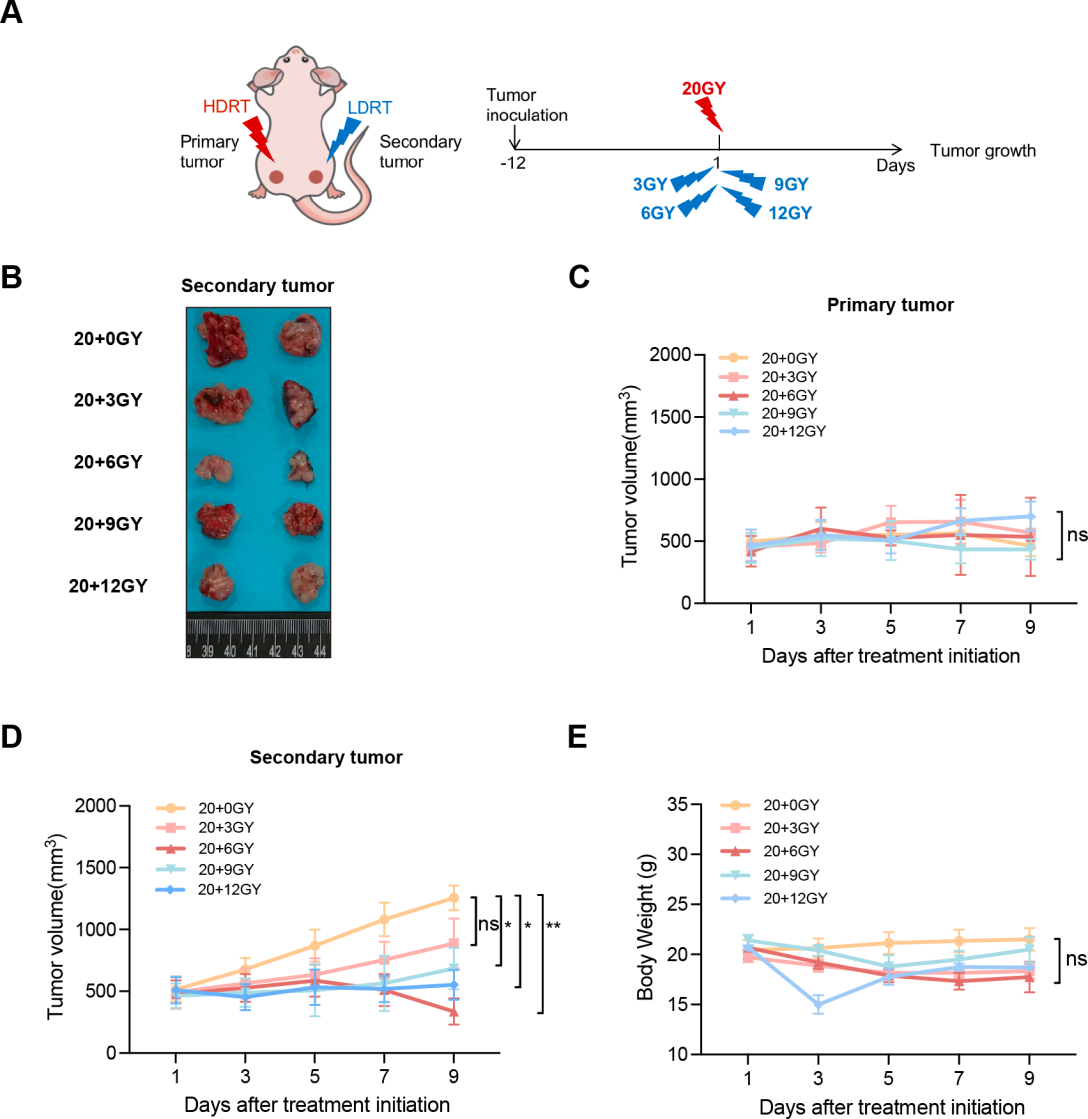
The combination of mixed dose radiation therapy mode inhibits tumor growth. **(A)** Treatment scheme. **(B)** Secondary tumor physical image. **(C, D)** In the mouse model constructed by LLC (n = 6-8 mice/group/time point, one-way analysis of variance), 4×10^6^ LLC cells were injected subcutaneously into the left hind limb to create the primary tumor, and an additional 4×10^6^ LLC tumor cells were implanted in the right limb to form the secondary tumor. The tumor volume was measured at intervals of one day commencing from seven days after tumor implantation. primary tumor **(C)** and secondary tumor **(D)** tumor growth curves. **(E)** Weight gain curve of irradiated mice. ns *P* > 0.05, *P < 0.05, **P < 0.01.

To investigate the effects of diverse irradiation doses on tumor growth inhibition and immune cell recruitment, we developed a bilateral LLC lung cancer model in C57BL/6J mice. To minimize radiation-induced damage to normal tissues, tumor cells were subcutaneously implanted into both lower limbs. The left limb received a uniform dose of 20 Gy, designated as the “primary tumor,” while the right limb was irradiated with varying doses of 3, 6, 9, or 12 Gy, termed the “secondary tumor” (**Figure 2A**). Tumor size was measured every other day following the initiation of irradiation. Our results demonstrated that the curative dose of 20 Gy effectively suppressed the growth of the “primary tumor” across all groups, with no significant differences in tumor size (**Figures 2B, C**). By day 9 post-irradiation, the average tumor volume in the “secondary tumor” groups receiving 3, 6, 9, and 12 Gy was reduced compared to the non-irradiated group, with the 20 + 6 Gy group exhibiting the most pronounced tumor inhibition (**Figures 2B, D**). To assess treatment-related toxicities, we monitored changes in mouse body weight and found no significant differences among the groups (**Figure 2E**). In conclusion, combined treatments using various irradiation dose regimens demonstrated inhibitory effects on tumor growth in mice, with the 20 + 6 Gy group exerting the most substantial influence. Notably, this treatment approach did not induce significant toxic side effects in the mice.

### The 20 + 6 Gy group demonstrates optimal RT efficacy

**Figure 3:**
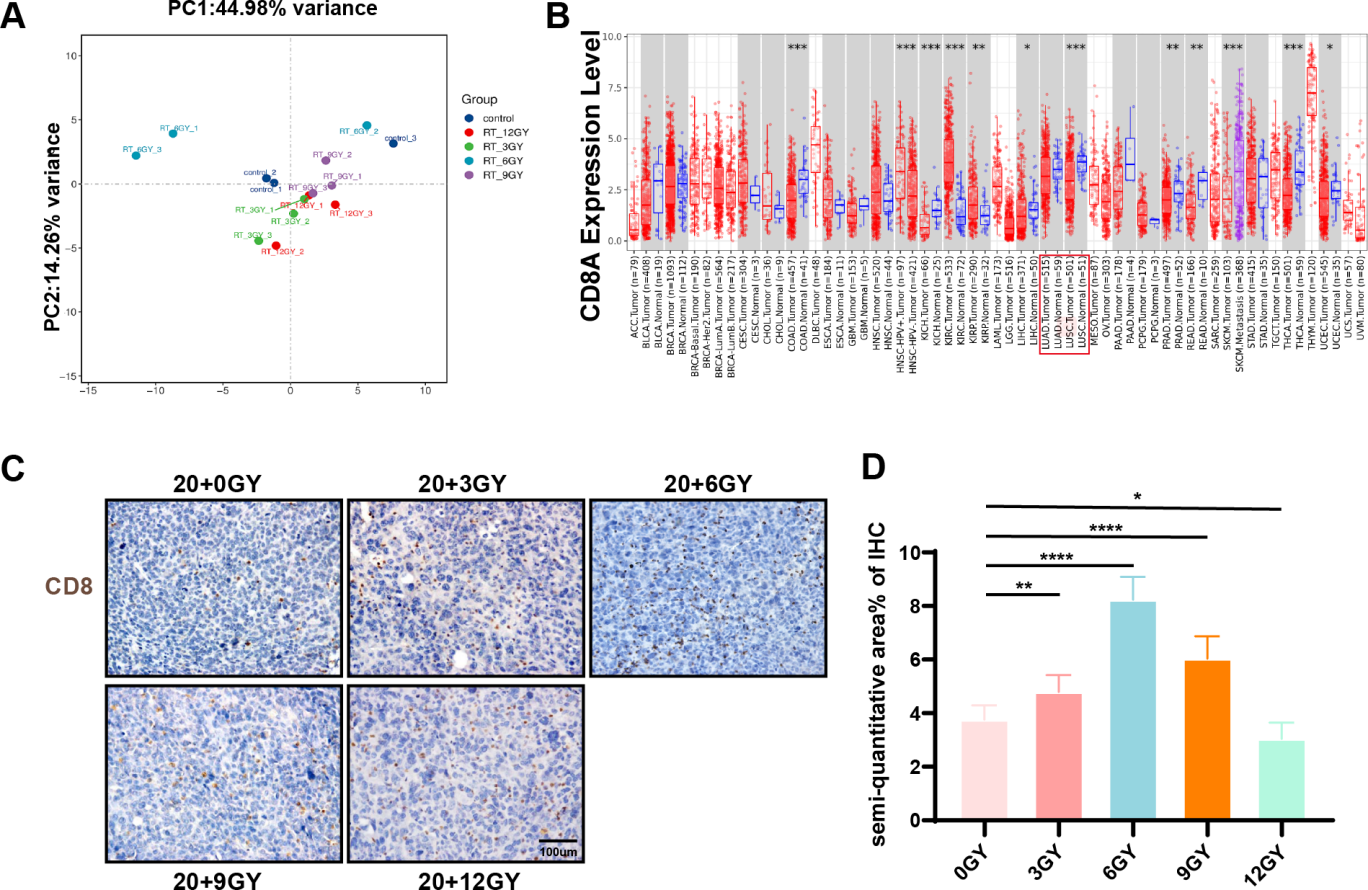
The 20 + 6 Gy group had the best radiotherapy mode. **(A)** Principal component analysis diagram. **(B)** Difference of CD8A expression levels in LUAD and LUSC in TIMER. **(C, D)** Immunohistochemical staining of CD8 in each group.To open an image in Image J, utilize the “Split Channels” feature to separate the image into three distinct files representing red, green, and blue channels. As hematoxylin staining of cell nuclei manifests as blue, we opt for the blue channel for color filtration. Navigate to the “Adjust” tab and select “Threshold.” The system will automatically outline the yellow-brown positively stained regions in red. Following appropriate adjustments based on the staining characteristics of each slice, apply the settings by clicking “Apply.” Ultimately, employ the “Analyze-Measure” function to derive the percentage of positively stained areas per unit area. *P < 0.05, **P < 0.01, ***P < 0.001,****P < 0.0001.

To investigate gene expression in the “secondary tumor” post-irradiation, we collected mouse tumor samples for RNA sequencing. Transcriptomic analysis was performed on the sequencing data, integrating gene detection values to generate expression box plots and conduct PCA (**Figure 3A**). While reproducibility across groups was generally satisfactory, occasional biased data points were observed. Consequently, we selected data points exhibiting reasonable repeatability between sample groups for subsequent analysis. Our findings revealed that the 20 + 6 Gy treatment regimen induced the most pronounced variations in gene expression within the “secondary tumor.” KEGG pathway enrichment analysis of DEGs across groups (**Supplementary Figure S5A**) indicated that the majority of these genes were primarily implicated in immune system functions. Given that CD8^+^ T cells are central to antineoplastic immunity, we focused our analysis on CD8 expression across various tumors. Using the TIMER database, we assessed CD8 expression between TCGA tumors and adjacent normal tissues. Notably, CD8 expression was significantly reduced in lung adenocarcinoma (LUAD) and lung squamous cell carcinoma (LUSC) tissues compared to adjacent normal tissues (**Figure 3B**). Additionally, CTLA4 expression was increased, while PD-L1 expression decreased in LUAD and LUSC tissues (**Supplementary Figures S5B, D**). CD8^+^ T-cell infiltration is frequently associated with favorable prognosis in solid tumors (34). Using the Kaplan-Meier plotter database, we investigated the correlation between these factors and lung cancer patient survival, finding that PD-L1 and CTLA4 did not significantly affect OS (**Supplementary Figures S5C, E**). Integrating transcriptomic analysis results, we performed pathway enrichment on immune system-related DEGs across groups (**Supplementary Figure S5F**). The 20 + 6 Gy group exhibited the highest number of upregulated differential genes. Further examination via the GEPIA database revealed that the majority of these upregulated genes were expressed at higher levels in normal lung tissues, suggesting that changes in the 6 Gy irradiated group might potentially improve lung cancer prognosis. Immunohistochemical staining for CD8 on tumor samples demonstrated that CD8^+^ T-cell infiltration was most pronounced in the 20 + 6 Gy group (**Figures 3C, D**). These findings confirm that the 20 + 6 Gy regimen represents the optimal combined irradiation dose for effectively recruiting CD8^+^ T cells.

### Combined treatment with 20 + 6 Gy irradiation and ICIs enhances tumor suppression and immune activation

**Figure 4:**
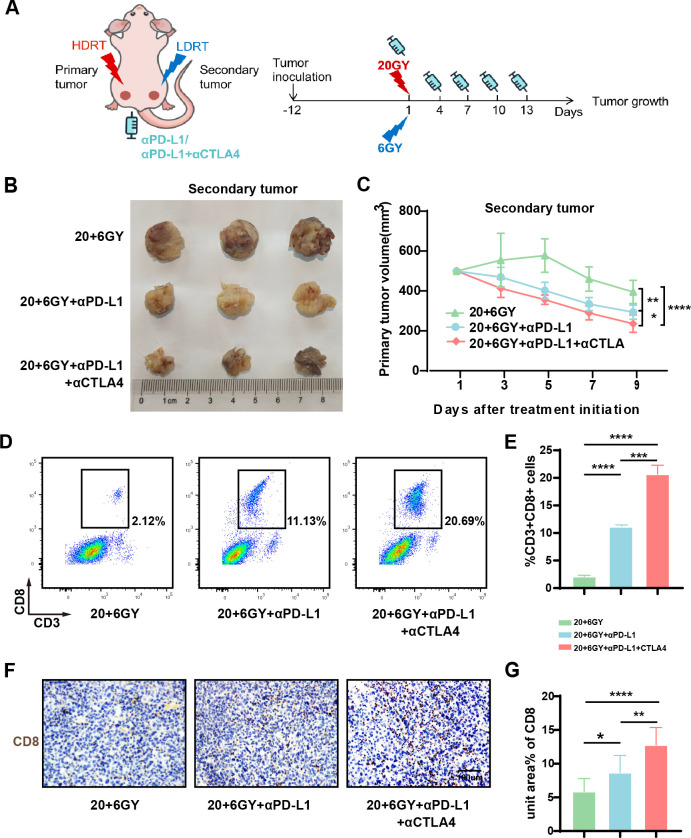
The combination of mixed dose radiation therapy mode combined with ICIs further inhibit tumor growth and activate the tumor immune microenvironment. **(A)** Treatment scheme. **(B)** Secondary tumor physical image. **(C)** Secondary tumor growth curve (n = 7-9 mice/group/time point, one-way analysis of variance). **(D, E)** Flow cytometry analysis of CD8^+^ T (CD3^+^CD8^+^ cells) in the tumors of each group (n = 3 mice/group, one-way analysis of variance); **(F, G)** Immunohistochemical maps of CD8 in the tumors of mice in each group. To open an image in Image J, utilize the “Split Channels” feature to separate the image into three distinct files representing red, green, and blue channels. As hematoxylin staining of cell nuclei manifests as blue, we opt for the blue channel for color filtration. Navigate to the “Adjust” tab and select “Threshold.” The system will automatically outline the yellow-brown positively stained regions in red. Following appropriate adjustments based on the staining characteristics of each slice, apply the settings by clicking “Apply.” Ultimately, employ the “Analyze-Measure” function to derive the percentage of positively stained areas per unit area.*P < 0.05, **P < 0.01, ***P < 0.001,****P < 0.0001.

To further validate the efficacy of combined RT and immunotherapy in tumor treatment, we incorporated two ICIs, anti-PD-L1 and anti-CTLA4, with RT in the 20 + 6 Gy group (**Figure 4A**). Nine days post-irradiation, the average tumor volume of the “secondary tumor” was significantly reduced in the 20 + 6 Gy+αPD-L1 group (293 mm³) and the 20 + 6 Gy+αPD-L1+αCTLA4 group (235 mm³) compared to the 20 + 6 Gy group (395 mm³) (**Figures 4B, C**). Notably, the combination of both ICIs resulted in more pronounced tumor suppression, with statistically significant differences observed. These findings demonstrate that LDRT combined with ICI treatment further enhances the inhibitory effect on mouse tumors without inducing apparent adverse effects. To assess tumor-immune infiltration, we analyzed CD8^+^ cell populations within CD3^+^ cells as characteristic markers for infiltrating T-cell populations. In the 20 + 6 Gy, 20 + 6 Gy+αPD-L1, and 20 + 6 Gy+αPD-L1+αCTLA4 groups, the percentages of CD8^+^ T cells were 2.12%, 11.13%, and 20.69%, respectively (**Figures 4D, E**). Immunohistochemical staining of mouse tumors corroborated these findings, revealing increased infiltration of CD8^+^ T cells in tumors treated with combined RT and ICIs compared to those treated with RT alone (**Figures 4F, G**). These results suggest that combined treatment with 20 + 6 Gy irradiation and ICIs can further enhance tumor inhibition and immune activation within the tumor microenvironment in mice.

### The 20 + 6 Gy irradiation regimen enhances intracellular CCL17 secretion and correlates with improved prognosis in NSCLC

**Figure 5:**
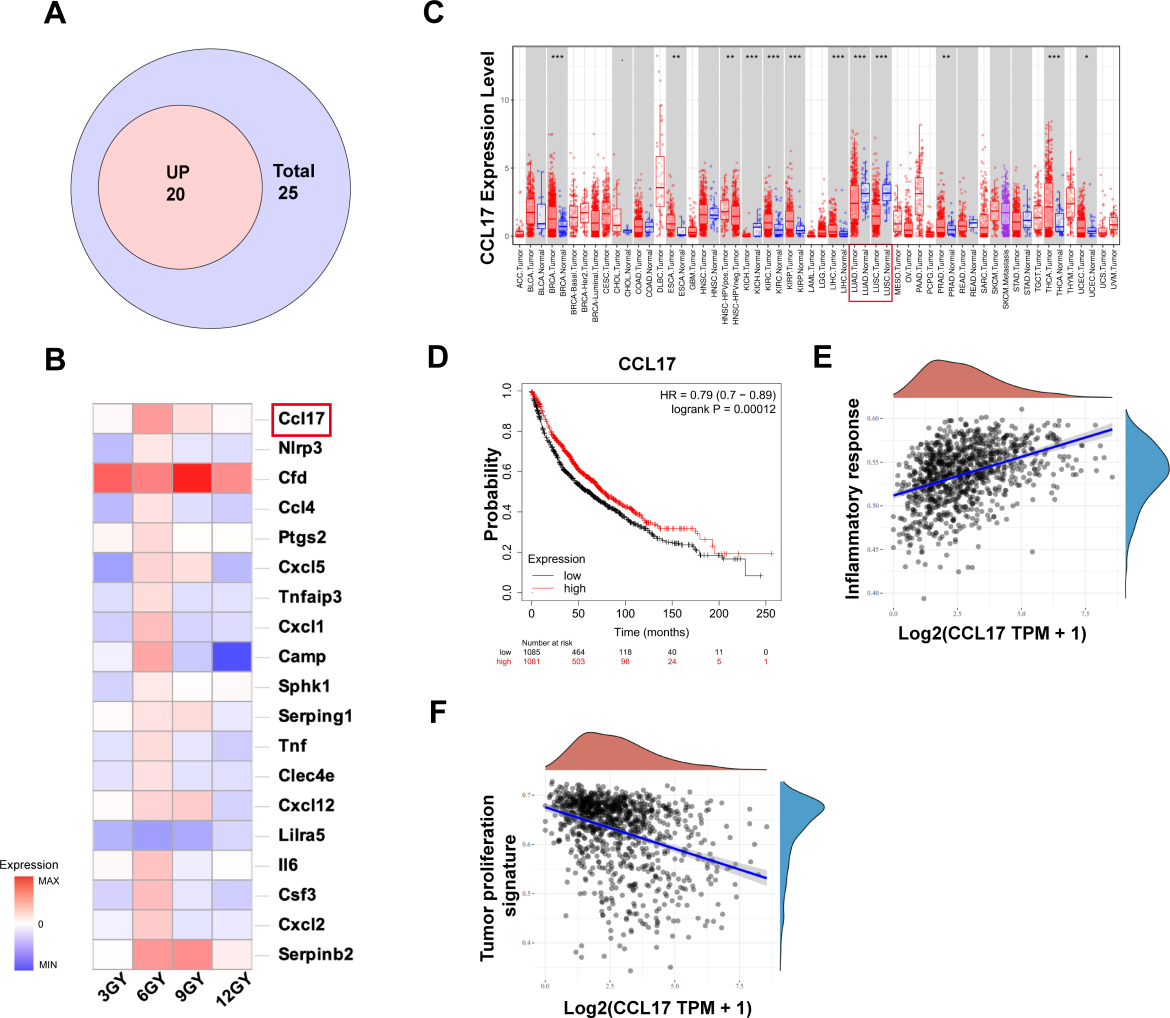
The combination of mixed dose radiation therapy mode increases intracellular CCL17 secretion, and its high expression is associated with good prognosis in non-small cell lung cancer. **(A)** The Venn diagram of the “up” gene. **(B)** Visualization analysis of differentially expressed genes in different dosage groups compared to the 20 + 0 Gy group. **(C)** Differences in the expression levels of CCL17 in LUAD and LUSC in IMER database; **(D)** Kaplan−Meier plotter database CCL17 and overall survival of patients with lung cancer were analyzed and progression-free survival curves. **(E)** Association of CCL17 with pro-inflammatory pathways in non-small cell lung cancer. **(F)** Correlation of CCL17 with pro-inflammatory pathways in non-small cell lung cancer. *P < 0.05, **P < 0.01, ***P < 0.001.

To elucidate the mechanism by which the 20 + 6 Gy group maximally activates the immune system, we conducted a comprehensive analysis of tumor RNA sequencing results. Among the DEGs related to the immune system in the 20 + 6 Gy group, 23 genes were upregulated and two were downregulated. We analyzed the expression of these genes in cancerous and adjacent normal tissues using the TIMER database, categorizing upregulated genes highly expressed in adjacent normal tissues as “up genes,” suggesting their potential to improve patient prognosis. Through rigorous screening, we identified 19 “up genes” (**Figure 5A**). Analysis of differences in expression levels across experimental and control groups (**Figure 5B**) revealed CCL17 as the most significantly upregulated gene in the 20 + 6 Gy group, prompting its selection as a potential target for antitumor effects in subsequent studies. Using the TIMER database, we analyzed CCL17 expression across all TCGA tumor types, focusing on the contrast between tumor tissues and adjacent normal tissues (**Figure 5C**). CCL17 expression was significantly lower in LUAD and LUSC tissues compared to adjacent normal tissues (p<0.0001). Investigation of the relationship between CCL17 expression levels and survival outcomes in lung cancer patients using the Kaplan-Meier mapper database demonstrated that high CCL17 expression was significantly associated with favorable prognosis (OS, HR=0.79 (0.70-0.89), p=0.00012) (**Figure 5D**). Furthermore, exploration of the correlation between CCL17 expression and key pathways in NSCLC patients (**Figures 5E, F**) revealed a positive correlation with pro-inflammatory pathways and a significant negative correlation with tumor proliferation pathways. These findings suggest that in patients with high expression and favorable prognosis, CCL17 likely exerts dual effects of promoting inflammation and inhibiting tumor proliferation.

### CCL17 promotes T-cell infiltration and activation in tumors

**Figure 6:**
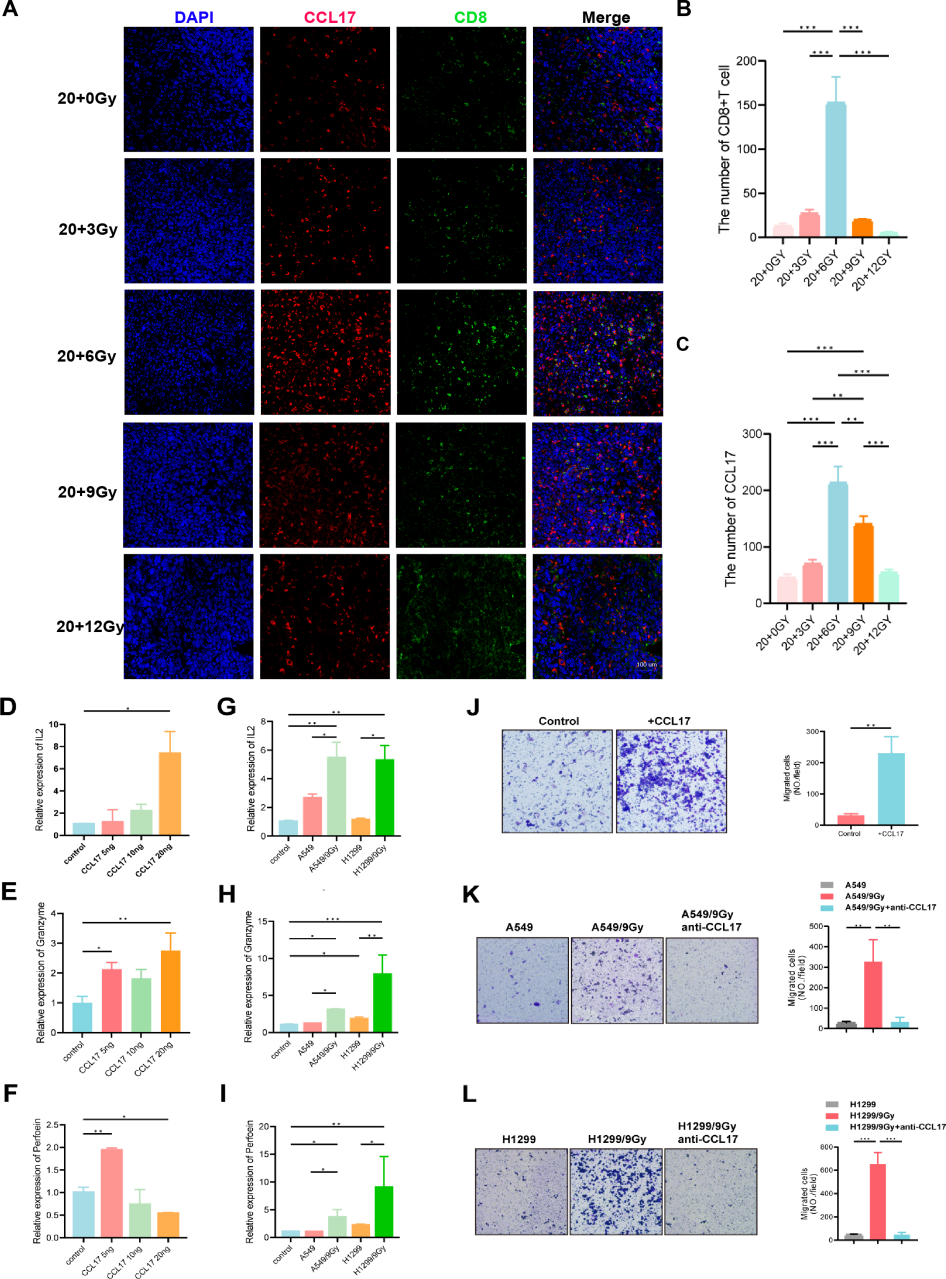
CCL17 promotes T-cell infiltration and activation in tumors. **(A-C)** Multiple immunofluorescence of CD8 and CCL17. **(D-F)** A total of 400,000 Jurkat T cells were inoculated into 6-well plates, and different concentrations of CCL17 cytokines were added to the medium. RNA was extracted after 72 h of culture. The T-cell activation indices IL-2, granzyme and perforin were determined by qPCR. **(G-I)** A total of 400,000 Jurkat T cells were inoculated into 6-well plates, and Jurkat T cells were cocultured with non-small cell lung cancer cells with different irradiation treatments. RNA was extracted 72 hours later. The T-cell activation indices IL-2, granzyme and perforin were determined by qPCR. **(J)** The migration ability of Jurkat cells was measured by adding CCL17 chemokines to the downward chamber. **(K, L)** The supernatant of A549/9G or H1299/9Gy cells was added to the lower chamber, and anti-CCL17 was added to detect the migration ability of Jurkat cells. *P < 0.05, **P < 0.01, ***P < 0.001.

When investigating the correlation between CCL17 expression and immune cell infiltration in tumors, initial analysis using the TISIDB database revealed a positive correlation between CCL17 expression and CD4 and CD8 expression (**Supplementary Figure S6A**). To validate these findings, we performed immunohistochemistry and multiple immunofluorescence staining for CD8 and CCL17 on tumor sections from the initial mouse group (**Figure 6A-C**, **3G**, **Supplementary Figure S6B**). The 20 + 6 Gy group showed the highest expression of CD8 and CCL17 compared to other groups, with an increased CD8^+^ T-cell population. These results suggest that increased CCL17 production may facilitate CD8^+^ T-cell infiltration in the tumor-immune microenvironment. We hypothesize that CCL17 exerts a chemotactic function on T cells within the tumor-immune microenvironment, attracting activated CD8^+^ T cells to the tumor site to elicit antitumor effects. Previous *in vivo* studies have demonstrated that CCL17 enhances T-cell infiltration into the tumor microenvironment. We subsequently validated these findings through *in vitro* experiments using two common NSCLC cell LLC. Irradiated progeny cells were exposed to doses of 3, 6, 9, and 12 Gy, and their survival was monitored (**Supplementary Figures S6C–E**). ELISA analysis of irradiated progeny cell supernatants revealed an overall increase in CCL17 secretion compared to unirradiated tumor cells. However, this increase peaked at a specific dose before decreasing, indicating that CCL17 expression is not linearly dependent on radiation dose. Notably, CCL17 levels in LLC supernatants peaked at 6 Gy, which is consistent with *in vivo* data, while A549 and H1299 cell supernatants showed peak CCL17 levels at 9 Gy. To investigate T-cell activation, Jurkat cells were cultured with varying concentrations of CCL17 cytokines. After 72 hours, qPCR analysis of T-cell activation markers (IL-2, granzyme, and perforin) revealed a significant increase in Jurkat cell activation, up to 7-fold, following CCL17 introduction (**Figures 6D-F**). Co-culture of Jurkat cells with irradiated and unirradiated lung cancer cells for 72 hours further enhanced T-cell activation (**Figures 6G-I**). Moreover, transwell assays demonstrated that CCL17 enhanced Jurkat cell migration (**Figure 6J**), while the addition of a CCL17 neutralizing antibody to A549/9 Gy and H1299/9 Gy supernatants inhibited this migration (**Figures 6K, L**).

To summarize, bioinformatic analysis revealed a positive correlation between CD4 and CD8 expression and CCL17. In addition, immunohistochemistry and multiple immunofluorescence studies demonstrated that increased CCL17 expression in tumors corresponded with increased CD8 infiltration. Further, *in vitro* studies confirmed substantial CCL17 chemokine secretion from precursor cells, which stimulated T-cell activation and migration.

## Discussion

Moore initially described systemic antitumor effects caused by radiation in 1953, associating them with stimulation of systemic immunity (35). However, distant reactions in clinical settings have been comparatively infrequent (26). Several factors, including radiation dosage, segmentation, chemotherapy, and the host’s immune microenvironment, appear to influence the induction of distant responses (36). Consequently, an appropriate RT combination and an associated regimen (i.e., radiation dosage and pattern) is required to achieve a successful response in distant areas. For combination therapy with different irradiation doses, we utilized an *in vivo* model of mouse lung cancer cells (LLC) to select the optimal combination dose. Our approach activates adaptive immunity, specifically cytotoxic CD8^+^ T cells, and potentiates systemic effects using ICIs like PD-L1 inhibitors and CTLA4 blockers.

Most lung cancer patients present with metastases at diagnosis, indicating advanced-stage cancer with a remarkably low 5-year survival rate (18, 19). High tumor load makes HDRT administration to all lesions impractical due to potential toxicity. For individuals with intact immune systems, applying relatively low radiation doses to small lesions may boost antitumor defenses (37). However, exactly how the doses should be combined has not been reported. Our data showed the most effective treatment involved 6 Gy irradiation. Wang et al. also found that 6 Gy irradiation upregulates tumor-infiltration molecules like intracellular adhesion molecule-1 (ICAM-1) or FAS in HCT-15 cells, supporting a possible synergistic enhancement effect of RT. In addition, 6 Gy irradiation has also been shown to activate the immune microenvironment in solid tumors (38). Typically, a cold tumor that has not been irradiated will exhibit a low density of activated CD8^+^ T cells and non-self CD8^+^ T cells in the primary tumor. By various means, we verified that the combined irradiation pattern of 20 + 6 Gy enhances CD8^+^ T-cell activation at the primary tumor site. In addition, we found that CCL17 expression was upregulated in 20 + 6 Gy irradiated mouse tumors. CCL17, a potent chemokine produced by thymus and antigen-presenting cells, binds to the CCR4 receptor and plays a crucial role in CD8^+^ T-cell function and migration (39). Interestingly, Ye et al. hypothesize that the OS of patients that show upregulated CCL17 expression is higher than patients with lower CCL17 expression, especially for patients with early (stages I and II) LUAD (40). Additionally, CCL17 can potentially act as an anticancer agent by introducing TILs into tumors (41). Valente et al. suggest that cross-talk between iNKT cells and CD8^+^ T cells in the spleen requires the IL-4/CCL17 axis for the generation of short-lived effector cells (42). In addition, other studies indicate that dendritic cell-produced IL-12 augments CD8^+^ T-cell activation through the production of the chemokines, CCL1 and CCL17. Taken together, these data reflect the pleiotropic role of CCL17 and its ligand, CCR4, in immune responses (43). By analyzing public databases, we show that tumors demonstrate decreased CD8^+^ T-cell densities and decreased CCL17 expression compared to normal tissues. In addition, we show that the mechanism by which tumors facilitate the involvement of non-natural CD8^+^ T cells and enhance tumor resistance following 6 Gy treatment is through the release of CCL17. Further investigations are required to determine the potential collaborative role of other factors in promoting the activation and migration of CD8^+^ T cells.

Recent immunotherapy research for NSCLC has shown progress, but low objective remission rates (ORRs) remain a drawback. First-line monotherapy achieves an ORR of 45%, whereas second-line therapy only results in a 20% ORR. In addition, immunotherapy carries a risk of hyper- (6%–29%) and pseudo progression (4.5%) (44). Earlier research on NSCLC has indicated that the use of ICIs along with RT improves the response to immunotherapy and extends the OS period (26). Our data further demonstrated that PD-L1 and anti-CTLA4 combined with RT significantly inhibited tumor growth. Despite this, the clinical applicability of this treatment strategy is yet to be determined.

In this study, we explored the effects of different radiation levels on the immune microenvironment through various dose combinations. Investigating different ways of boosting the immune system in cancer is crucial for minimizing radiation-induced harm and enhancing immunotherapy efficacy. Nonetheless, the uniform radiation distribution observed in this work is somewhat tedious, and forthcoming research may favor the use of aggregate accumulated dosage of multi-fraction treatment. Importantly, our study was limited to animal experiments, and future clinical trials are crucial. Further investigation is required to evaluate the frequency of this reaction in alternative cancer models and to ascertain the respective functions of the natural and adaptive immune systems in these impacts.

## Data Availability

The raw data supporting the conclusions of this article will be made available by the authors, without undue reservation.
