# Supplementary figures and images for: Mixed radiation with different doses induces CCL17 to recruit CD8+T cell to exert anti-tumor effects in non-small cell lung cancer
Source: Front Immunol. 2025 Jan 14;15:1508007. doi: 10.3389/fimmu.2024.1508007 (PMC11772420; doi:10.3389/fimmu.2024.1508007)

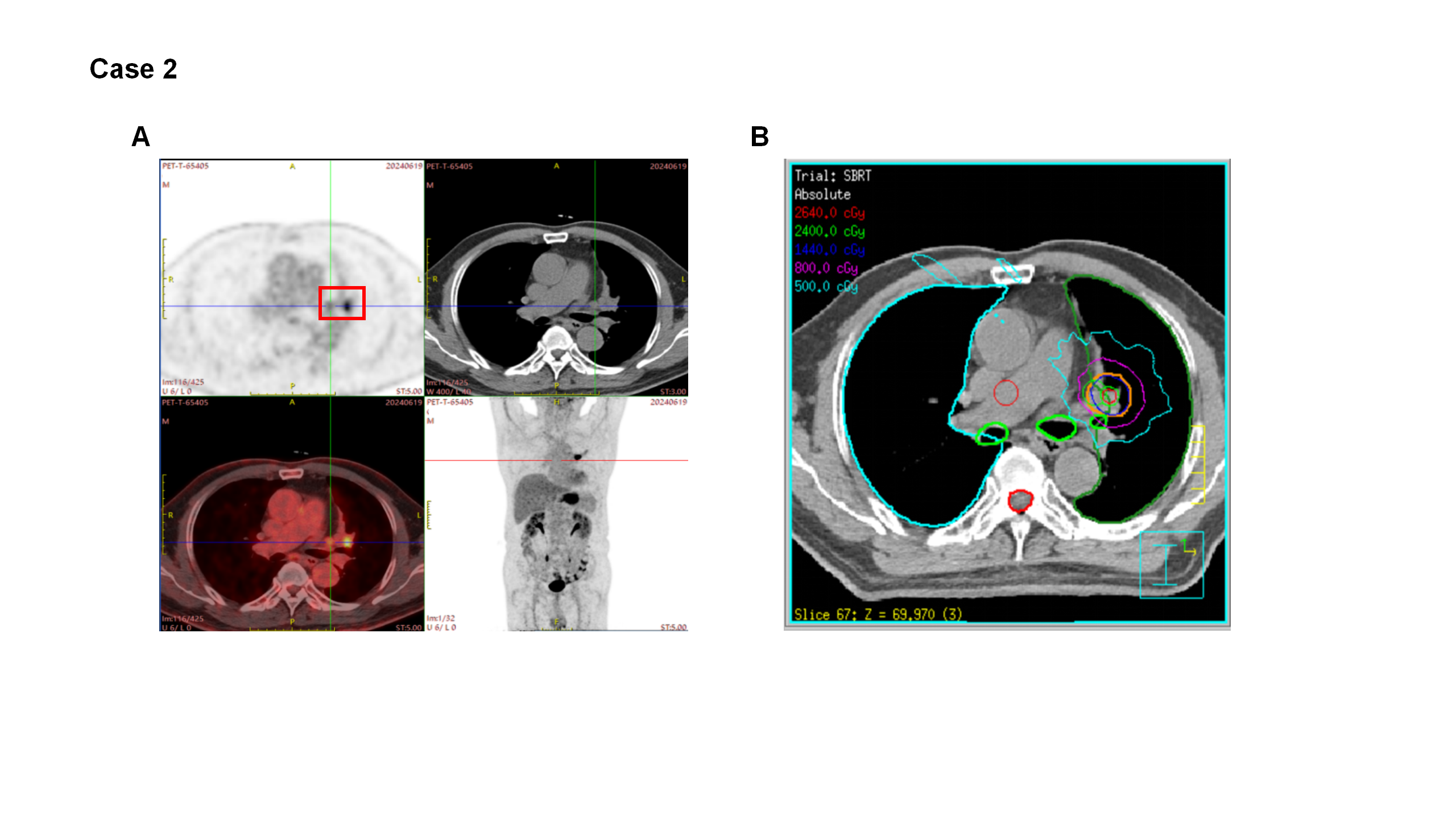

Supplement: Supplementary file 1 [file Image1.tif]

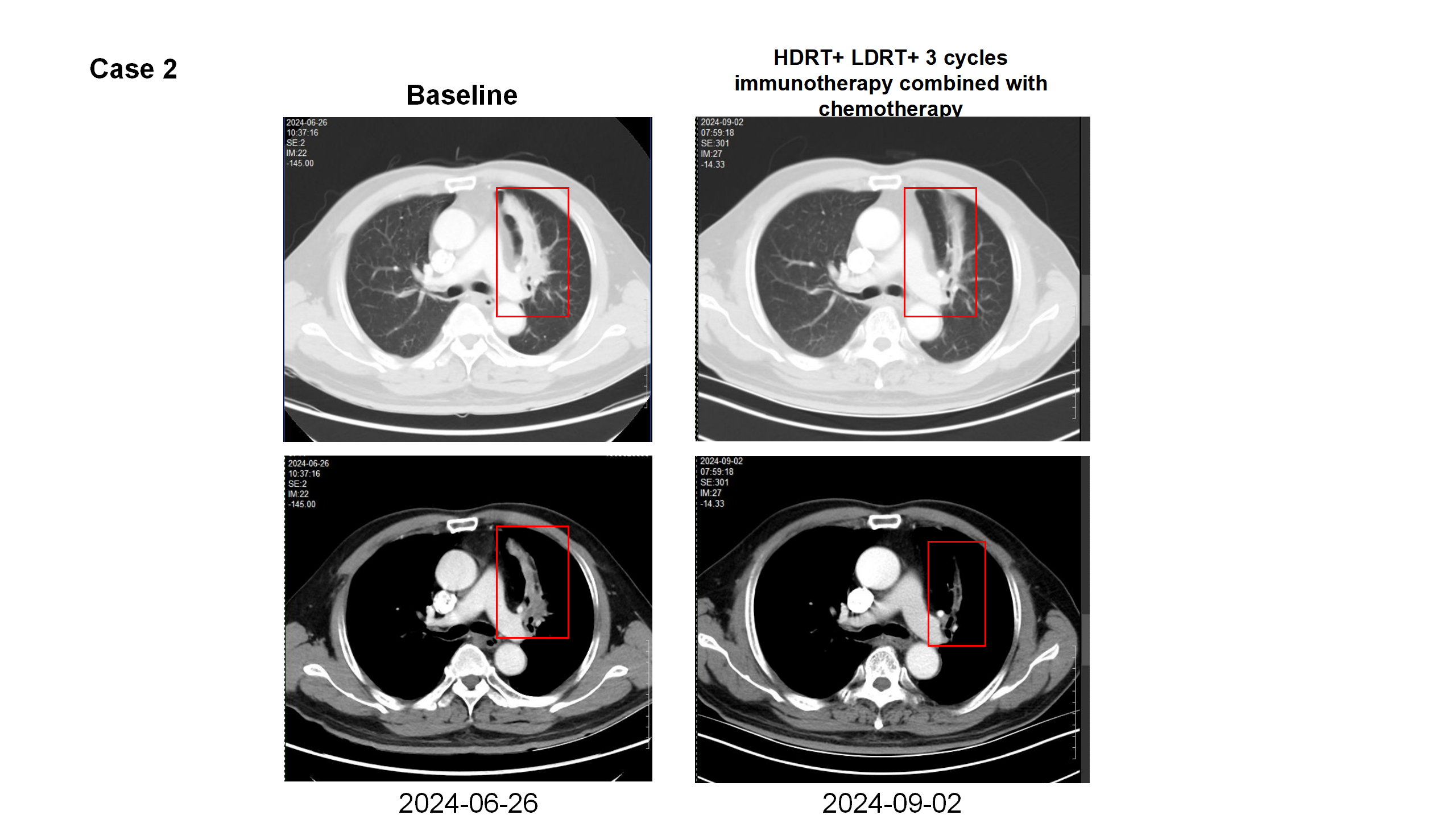

Supplement: Supplementary file 2 [file Image2.tif]

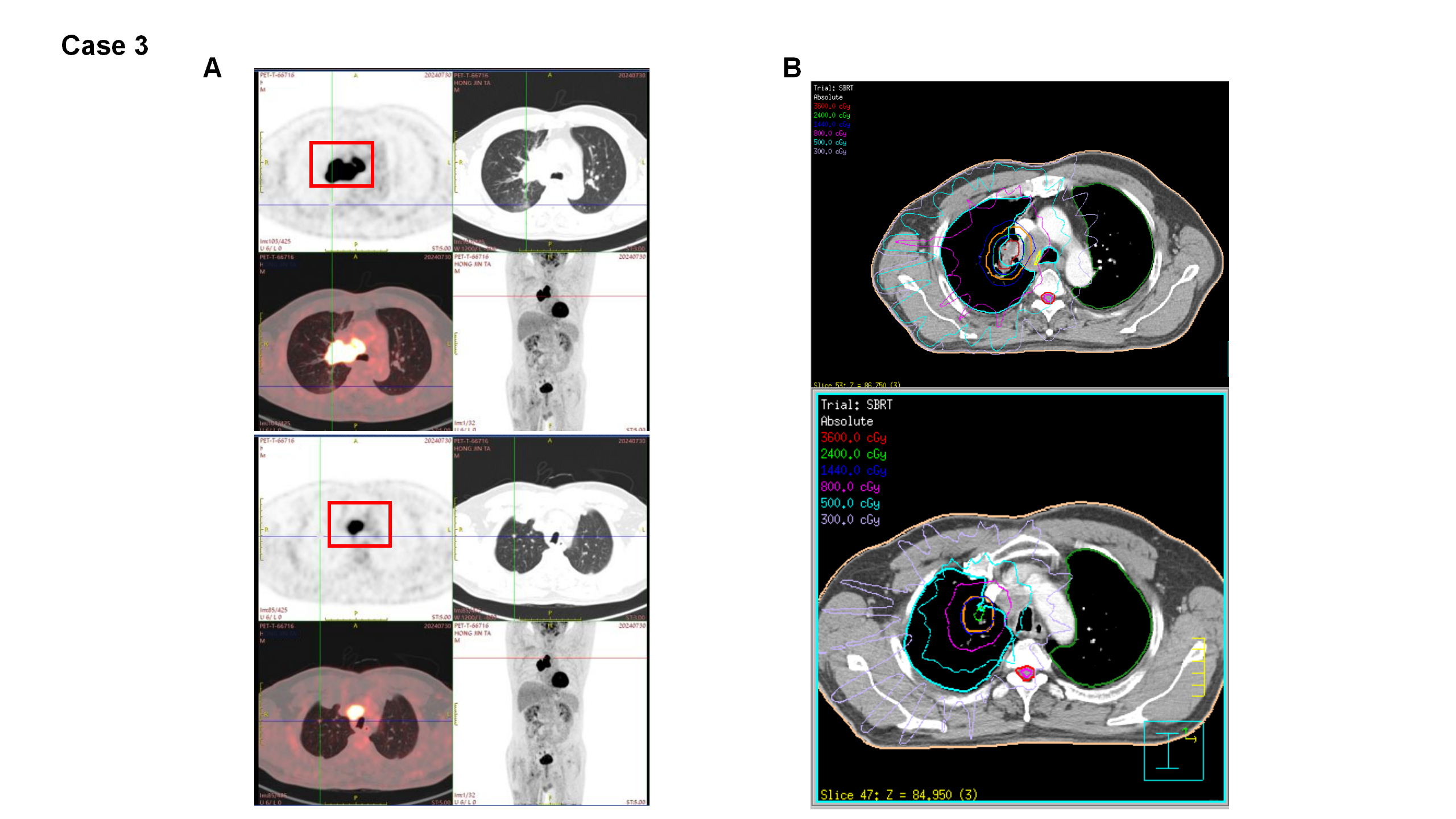

Supplement: Supplementary file 3 [file Image3.tif]

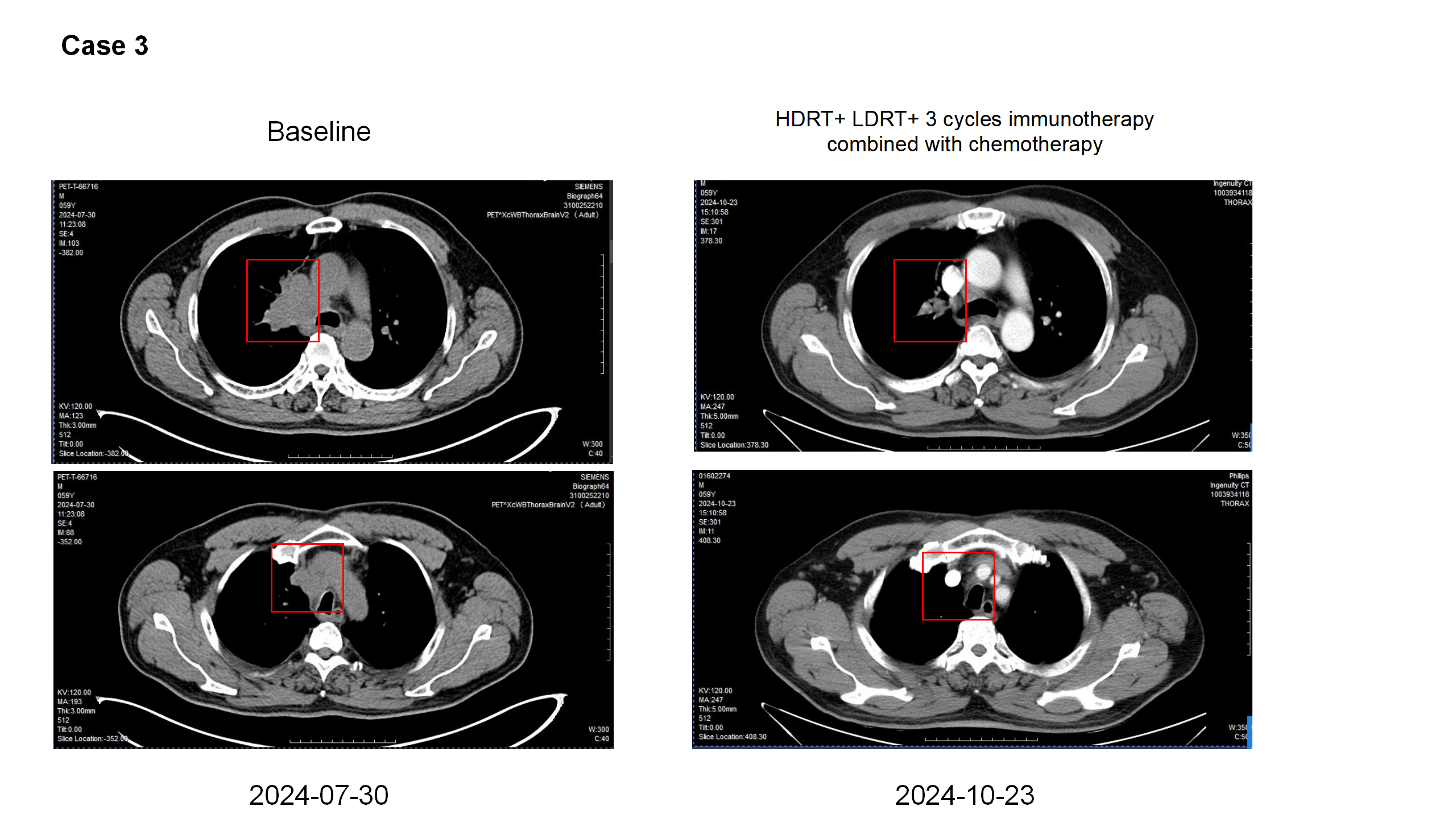

Supplement: Supplementary file 4 [file Image4.tif]

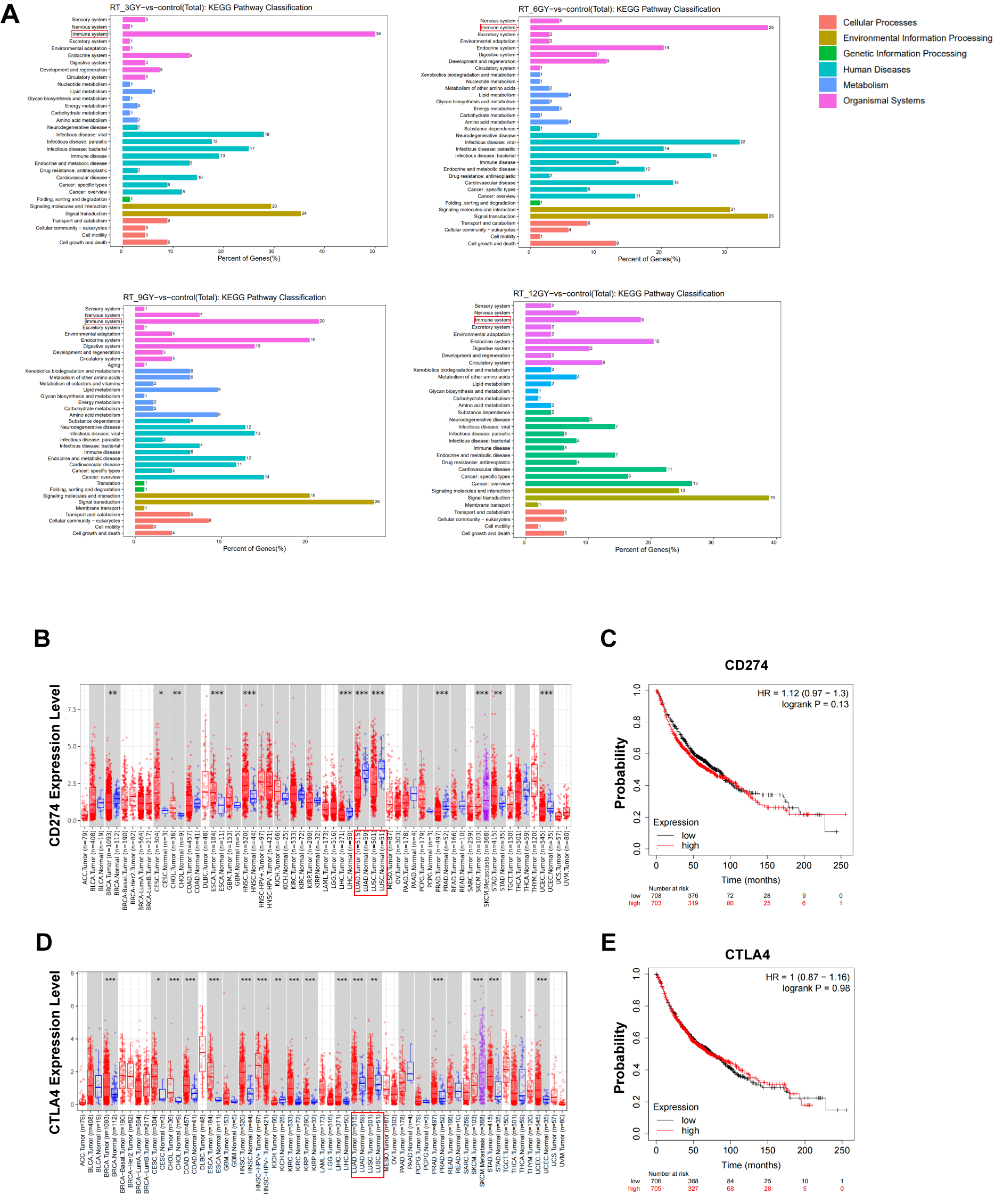

Supplement: Supplementary file 5 [file Image5.tif]

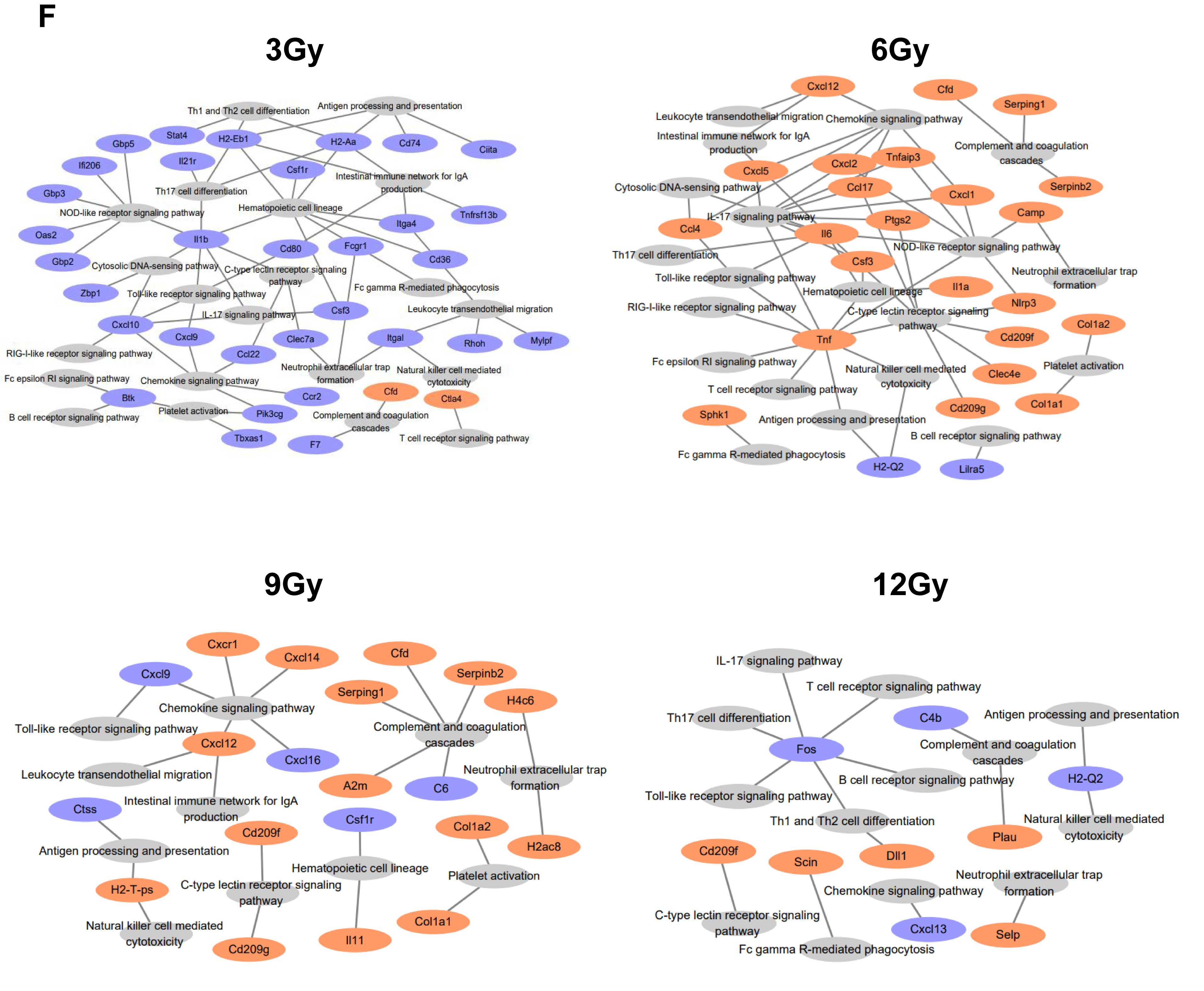

Supplement: Supplementary file 6 [file Image6.tif]

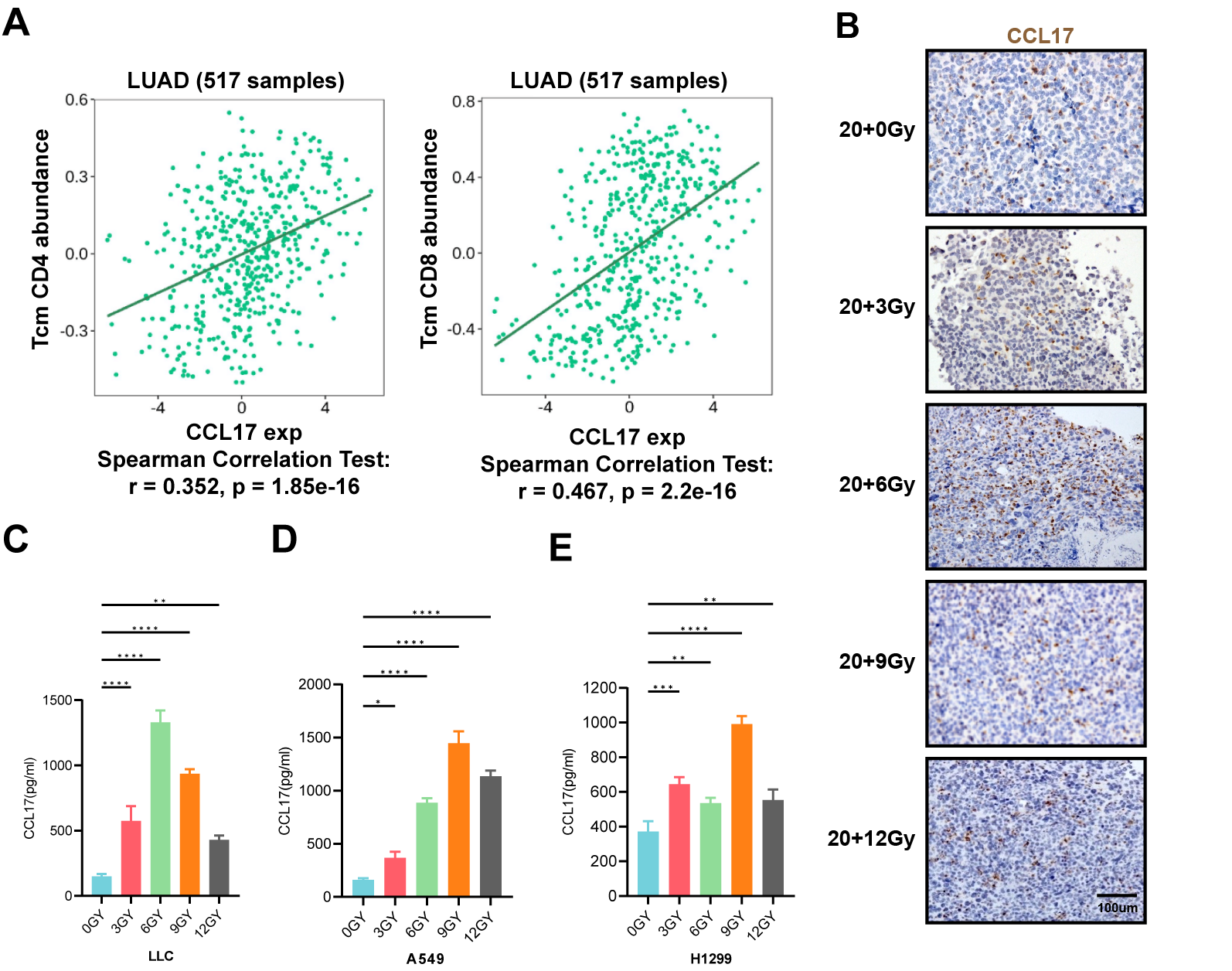

Supplement: Supplementary file 7 [file Image7.tif]
