# Supplementary material for: Mixed radiation with different doses induces CCL17 to recruit CD8+T cell to exert anti-tumor effects in non-small cell lung cancer
Source: Front Immunol. 2025 Jan 14;15:1508007. doi: 10.3389/fimmu.2024.1508007 (PMC11772420; doi:10.3389/fimmu.2024.1508007)
Supplement: Supplementary file 8 [file DataSheet1.docx]

Supplementary Material

# Supplementary Figures and Tables

## Supplementary Figures

**Supplementary FIGURE S1. Typical PET-CT Images and Radiotherapy Strategy for Case 2 at Initial Diagnosis**

(A) PET-CT showed increased FDG uptake in the upper left lung lobe and left hilar lymph nodes (marked with a red border). (B) The lesion in the upper left lung lobe received SBRT with a prescribed dose of 24Gy / 3F, and the adjacent left hilar lymph nodes (cyan area) received a lower dose of 5Gy.

**Supplementary FIGURE S2. CT Images of Case 2 from Baseline to Recent Assessment**

Compared to the baseline, CT scans after SBRT combined with three cycles of chemotherapy and immunotherapy showed regression of the lesion in the upper left lung lobe and alleviation of atelectasis (marked with a red border).

**Supplementary FIGURE S3. Typical PET-CT Images and Radiotherapy Strategy for Case 3 at Initial Diagnosis**

(A) PET-CT imaging showed increased FDG uptake in the upper right lung lobe and right upper paratracheal lymph nodes (highlighted with a red border), indicating the presence of malignant tissue. (B) The radiotherapy strategy for Case 3 involved delivering SBRT to the lesion in the upper right lung lobe with a prescribed dose of 24 Gy in 3 fractions (24 Gy/3F). The majority of the adjacent right upper paratracheal lymph nodes (indicated by the cyan area) received a low dose of 5 Gy, while the more distant cephalic portion of the lesion only received a dose of 3 Gy (indicated by the lavender area).

**Supplementary FIGURE S4. CT Images of Case 3 from Baseline to Recent Assessment**

CT scans taken at baseline and after SBRT combined with three cycles of chemotherapy and immunotherapy showed partial remission of the tumor lesion in the upper right lung lobe and right upper paratracheal lymph nodes(highlighted with a red border).

**Supplementary FIGURE S5.**

(A) KEGG pathway enrichment analysis. (B) Differences in CD274 expression levels in different tumors in the TIMER database; (C) Survival curve of CD274 in the Kaplan‒Meier database. (D) Differences in CTLA4 expression levels in different tumors in the TIMER database. (E) Survival curve of CTLA4 in the Kaplan‒Meier database; (F) Pathway enrichment of immune system-related differential genes (orange for up-regulated differential genes in the immune system, blue for down-regulated differential genes). **P* < 0.05, ***P* < 0.01, ****P* < 0.001.

**Supplementary FIGURE S6.**

1. Correlation between CCL17 and CD4 and CD8 expression in lung adenocarcinoma. (B) Immunohistochemistry of CCL17. (C) Secretion of CCL17 in LLC after different doses of irradiation. (D) Secretion of CCL17 in A549 cells after different doses of irradiation. (E) Secretion of CCL17 in H1299 cells after different doses of irradiation. **P* < 0.05, ***P* < 0.01, ****P* < 0.001.

## Supplementary Table

**Sequence of primers.**

| Name |  | Sequence |
| --- | --- | --- |
| perforin-F |  | GGCTGGACGTGACTCCTAAG |
| perforin-R |  | CTGGGTGGACGCCTTGAAG |
| Granzyme B-F |  | CCCTGGGAAAACACTCACACA |
| Granzyme B-R |  | GCACAACTCAATGGTACTGTCG |
| IL2-F |  | ACCTCAACTCCTGCCACAAT |
| IL2-R |  | TCCTGGTGAGTTTGGGATTC |
| β-actin-F |  | AGTGTGACGTGGACATCCGCAAAG |
| β-actin-R |  | ATCCACATCTGCTGGAAGGTGGAC |
